# Long-term effects of conservative treatment of Milwaukee brace on body image and mental health of patients with idiopathic scoliosis

**DOI:** 10.1371/journal.pone.0193447

**Published:** 2018-02-23

**Authors:** Ewa Misterska, Jakub Głowacki, Maciej Głowacki, Adam Okręt

**Affiliations:** 1 Department of Pedagogy and Psychology, University of Security, Poznan, Poland; 2 HCP Medical Centre, Poznan, Poland; 3 Department of Pediatric Orthopaedics and Traumatology, Poznan University of Medical Sciences, Poznan, Poland; University of Illinois at Urbana-Champaign, UNITED STATES

## Abstract

We aimed to provide a complex assessment of adult females with adolescent idiopathic scoliosis (AIS) after a minimum of 23 years after completed Milwaukee brace treatment. In the present study, a comparison between healthy female and AIS patients’ perception of trunk disfigurement, self-image, mental health, pain level and everyday activity was made. Thirty AIS patients with a mean of 27.77 yrs (SD 3.30) after the treatment were included in the study. The control group consisted of 42 females, matching the age profile of the patient group. Study participants from both groups were examined using the same protocol, except for the radiological evaluation. Patients and healthy controls completed the Polish versions of the Scoliosis Research Society (SRS-22) and Spinal Appearance Questionnaire (SAQ). Patients additionally filled the Bad Sobberheim Stress Questionnaire-Deformity (BSSQ-Deformity) and Bad Sobberheim Stress Questionnaire-Brace (BSSQ-Brace). The study group’s SAQ results differ significantly in regard to the total score and all individual domains, indicating better functioning among healthy controls. Except for the General domain (p = 0.002), among the remaining subscales the study group’s results differed significantly at p<0.001. Considering SRS-22 results, it was revealed that the patient group scored higher, signaling better functioning with reference to pain level (p = 0.016), function/activity (p<0.001) and the total score (p<0.001). The findings add to the complexity of long-term effect evaluations of AIS, particularly amongst females treated with a Milwaukee brace. Long-term results were not conclusive in terms of nonverbal assessment of body image and emotional tension regarding the experiences of brace-wearing. Future patients can be reassured that scoliosis treated conservatively does not negatively affect everyday activity, pain level, childbearing and mental health. Subjects who declared to have psychological problems due to scoliosis had a bigger curve size after treatment and in this study than the other AIS patients.

## Introduction

A number of studies have been conducted to investigate a long-term follow-up after surgical or conservative treatment for adolescent idiopathic scoliosis (AIS), concerning different types of brace treatment, among other subjects [1–7]. Danielsson and Nachemson [8] indicated that adults with scoliosis might be further concerned about their appearance, leading to some restriction of social and sexual activity. On the other hand, Noonan et al. [9] showed that, after reaching adulthood, there were no differences of a psychosocial nature in patients treated for AIS compared with healthy controls. It must be emphasized, most studies with long-term results regarding AIS refer to patients treated surgically [10–13].

The Milwaukee brace has been a standard of nonsurgical treatment for scoliosis since 1954 [14]. It was indicated that wearing the Milwaukee brace for 23 hours was the most effective treatment method for moderate scoliosis. Despite the effectiveness, a study conducted by Climent et al. concerning the impact of the type of brace on the health-related quality of life (HRQoL) of adolescents with spine deformities showed that the Cervico-Thoraco-Lumbosacral Orthosis (CTLSO) (e.g. Milwaukee brace) leads to a significantly greater impairment of the patient’s functioning during treatment than other types of orthoses [15,16]. Interestingly, Apter et al. [17] investigated the psychosocial sequelae of treatment using the Milwaukee brace in females with AIS and found they were able to cope well after an initial critical period. The authors indicated that only minor disturbances to body image and sexual attitudes were observed and, in general, no specific psychiatric intervention was needed in those patients [17]. In addition, Gratz et al. [18] suggested that after the initial shock of learning about the condition and treatment, the negative effects of the Milwaukee brace-wearing experience were minimal. The experience of "being different" was reported to be constructive by 12.5% of the patients. The negative aspects reported by the study group were related to buying clothing, limited movement, and the rudeness shown toward them by others. Meanwhile, Maruyama et al. [14] adopted part-time wearing of the Milwaukee brace in order to maintain its effectiveness and at the same time to reduce the physical and psychological burden on the patients. Their findings indicated that this was effective and did not affect the patients' HRQoL [14].

Unfortunately, some of the studies mentioned above had shortcomings, such as patients not being precisely defined nor consecutively selected or the lack of a comparison group consisting of healthy controls, which may restrict the generalizability of the findings [1–5, 19]. Moreover, most of research concerning the long-term results of conservative treatment concerns patients treated with the Boston brace [20–22].

To date, none of the mentioned studies have used validated questionnaires for nonverbal assessment of the perception of trunk disfigurement or scoliosis-specific stress connected to it and experiences related to wearing the Milwaukee brace. Taking into account the unambiguous results of a significant long-term psychological impairment related to conservative treatment, we aimed to provide a complex assessment of adult females with AIS minimum 23 years after completed Milwaukee brace treatment. In the present study, a comparison between healthy females' and AIS patients’ perception of trunk disfigurement, self-image, mental health, pain level and everyday activities was performed. Our hypothesis was that significant differences between these groups would be confirmed, indicating better psychosocial adaptation in the healthy controls. Furthermore, we aimed to evaluate patients’ memories of brace- and deformity-related emotional stress levels. We hypothesized that most of the patients would indicate a moderate or severe stress level, and that Milwaukee brace-related stress would be higher than the emotional tension related to body deformity. The last purpose was to identify socio-demographic and clinical factors affecting patients’ functioning, with the hypothesis that there is no or a weak association between radiological and clinical data and patients’ well-being. We have achieved the study objectives that have been set.

## Material and methods

### Structure of the study

In the present study, results concerning the implications of brace treatment in adult AIS females (scoliosis group-SG) treated with a Milwakee brace were evaluated. Based on an extensive search of Pediatric Orthopedics and Traumatology Clinic charts, we retrospectively reviewed the clinical records and radiographs of all female patients who had successfully completed a course of treatment with the Milwaukee orthosis between 1974 and 1990. Forty patients met the criteria for inclusion, but due to a change in personal details (such as address or family name after marriage), not all of them were contacted. Finally, 30 women participated in the evaluation.

A control group of healthy females (healthy controls group-HG) was selected for comparison based on random sample choice. The study and control group have been tested for equivalence in regard to their size and the quantitative and qualitative characteristics.

The groups were interviewed for age, work, marital status, number of children and how they were delivered, rate of caesarian sections and complications during delivery, place of residence and active hobby. In addition, all study participants were asked to fill in questionnaires to compare long-term brace-wearing's psychosocial implications.

All study participants were examined using the same protocol, except for the radiological evaluation performed in scoliosis patients only. They were informed in detail on the objective of the study. They understood that they would be anonymous and that their personal information would not be disclosed. All participants signed written informed consent to participate in the study. The study design was approved by the Bioethics Comission of Poznan University of Medical Sciences and was carried out in accordance with universal ethical principles.

### Clinical and radiological examination

Clinical and radiological examinations were performed at three time points: before, after completed treatment and then in the current follow-up and were taken in an upright position with the iliac ala exposed in an anterior-posterior projection. Data concerning former treatment regimens and radiological findings were gathered from a chart and radiograph review. The physical examinations were performed by AO, the 4^th^ study author, and the radiographic measurements were conducted by JG and MG, the 2^nd^ and the 3^rd^ study authors, respectively.

The success rate at maturity was calculated according to Nachemson and Peterson, who defined success of treatment as an increase in the curve of less than 6° from the start of bracing [23]. The curve change from end of treatment to the present follow-up was assessed as well.

### Patient sample

Thirty AIS patients with a minimum of 23 years after completed Milwaukee brace treatment were included in the study. All treatments were completed before the patients reached 19 years of age. In all cases, the scoliosis was not detected before 10 years of age and was not combined with any major spine deformities at the time when brace treatment was implemented. In addition, patients were excluded from the study if at the present study they suffered from any other disease leading to trunk deformity.

### Healthy controls

The control group consisted of 42 females, matching the age profile of the patient groups. The exclusion criteria for the control group were: previous back surgery or significant scoliosis, which was ruled out by clinical examination, including the use of the Perdriolli’s scoliometer. None of the controls had a trunk rotation of more than 5°, according to Danielsson et al. [7].

### Questionnaires used in this study

To capture the impact of the disease and its treatment, suitable and specific questionnaires were selected. Patients and healthy controls completed the Polish versions of Scoliosis Research Society (SRS-22), and Spinal Appearance Questionnaire (SAQ). In addition, to assess memories of brace- and deformity-related stress experience, only patient group was additionally asked to fill in the Bad Sobberheim Stress Questionnaire-Deformity (BSSQ-Deformity) and Bad Sobberheim Stress Questionnaire-Brace (BSSQ-Brace), which directly concern AIS or the use of braces [24–26].

*SRS-22* is a well-recognized self-assessment instrument used in the clinical evaluation of patients with AIS, which reflects the subjective perception of the patient’s health and measures health-related quality of life (HRQoL) [27]. SRS-22 contains 22 questions, which are grouped to form the following subscales (domains): intensity of pain; self-image; function/activity; mental health and satisfaction from treatment. The scores for each answer range from 1 to 5 points and in each domain the recipient can score from 5 to 25 points, except for the satisfaction from treatment subscale, on which patients can score from 2 to 10 points. The overall score can range from 22 to 110 points. However, the mean values in each domain are usually analyzed [28]. Higher values indicate better patient functioning.

*BSSQ-Deformity and BSSQ-Brace* consider specific requirements related to the necessity of conservative treatment and stress related to body deformation, connected with AIS. BSSQ-Brace and BSSQ-Deformity have a very similar structure and both consist of eight questions. BSSQ-Deformity relates to the effect of spine deformity on patients’ mood, interactions with their social environment and, as a result, the effect of the experienced stress. The BSSQ-Brace focuses on the psychological burden connected with the necessity of conservative treatment and assesses the extent to which brace wearing affects mood, distorts social interactions and, in consequence, leads to an increase in the stress level [29,30]. Possible answers on the Bad Sobberheim Stress Questionnaires are marked on a four-point scale: from 0 to 3; general scores range from 0 to 24. The results are interpreted as follows: the higher the score, the lesser the stress, thus 0 signifies the greatest stress, whereas 24 signifies the least stress. The following subdivision of the score values is proposed by Botens-Helmus et al.: 0–8 (strong stress), 9–16 (moderate stress) and 17–24 (little stress) [29].

The *SAQ*, a modified version of the Walter Reed Visual Assessment Scale (WRAS), is used to assess the perception of trunk deformity by scoliosis patients [31]. It comprises of trunk profiles depicting various degrees of trunk deformity caused by scoliosis, as included in the WRAS scale. Moreover, the SAQ includes close-ended questions, pertaining to the degree patients’ satisfaction or dissatisfaction with their appearance. To summarize, the SAQ consists of 20 items which form the following subscales that reflect various forms of body deformity: general, curve, prominence, trunk shift, waist, shoulders, kyphosis, chest and surgical scar (this domain was omitted in our analyses). The items are scored from 1 to 5 points. The higher the score, the worse the patients’ perception of appearance. Items no. 8, 18 and 20 are open-ended questions that focus on which aspect of deformity is the most bothersome to patients [31].

### Statistics

With respect to the statistical quantitative (numerical) features, e.g. age, apical translation, Cobb angle, number of children or questionnaire results, we determined the mean, 95% confidence intervals, range and standard deviations. Regarding the qualitative features, (information that has aspects that are impossible to be measured), e.g. curve type, educational level, marital status or place of residence, we gave the number of units that belong to described categories of a given feature and respective percentages. To determine if the investigated sample sizes were equivalent, the chi-square test was used. The chi-square test was used to compare qualitative features between persons with scoliosis and healthy controls. In addition, a Mann-Whitney test was utilized to compare differences between both groups in regard to quantitative characteristics. To establish relations between quantitative data such as e.g. age, duration of brace application, apical translation, Cobb angle, and questionnaire results, we used Spearman's rank correlation (marked as rS). To determine dependency between quantitative and qualitative characteristics, e.g. between questionnaire numerical data and marital status, place of residence or curve type, ANOVA Kruskal-Wallis test was used. To protect against Type I errors, a Bonferroni adjustment for multiple comparisons was made in that way the accepted alpha level (p = 0.05) was divided by the number of tests conducted in each section.

As the border level of statistical significance, we adopted p = 0.05; test results whose p value exceeded this level were treated as insignificant. For test results whose p value did not exceed the level of 0.05, effect size (ES) was calculated by means of Cramer's V or Glass's Δ.

Statistical calculations were performed by Statistica software. calculations were performed by Statistica software. See the supplementary material file containing clinical, radiological, socio-demographic and questionnaire data. See the supplementary material file containing clinical, radiological, socio-demographic and questionnaire data.

## Results

### Clinical and radiological data

The sample sizes are equivalent (p = 0.157). The patients’ mean follow-up period was 27.77 yrs. SD 3.30 (range 23–35 years). The Milwaukee brace was worn for a mean of 22.9 hrs. daily SD 0.31 (range 22–23). The average length of brace application was 45.47 months SD 20.00 (range 24–104).

Radiographic examination at the beginning of brace treatment resulted in Risser Grade 0 in 19 patients (63.33%), Risser Grade I in 2 patients (6.67%), and Risser Grade II in 9 patients (30%). Risser Grade IV was identified after completed treatment in all study patients (100%). In accordance with the criteria of the Scoliosis Research Society regarding the location of apex [32], thoracic scoliosis was identified in 21 patients (70%), thoracolumbar in 2 patients (6.67%) and lumbar curves were identified in 7 AIS females (23.33%). The success rate at maturity, according to Nachemson and Peterson [23], was identified in 16 patients (53.33%). Five patients (16.67%) were qualified for scoliosis surgery after completed brace treatment, but refused to undergo an operation. The curve change from the end of treatment to the present study was 9.1 angles SD 7.64 (range 0–27). For additional clinical and radiological characteristics of the patient group, see Table 1.

**Table 1:** Clinical characteristics of patients.

Characteristics	Mean (SD)	Range	N (%)
Brace application [hours/day]	22.9 (0.31)	22–23	---
Brace application [months]	45.47 (20.00)	24–104	---
Time after completed treatment [years]	27.77 (3.30)	23–35	---
Body Mass Index			---
Before treatment	17.47 (3.78)	13.64–22.83	---
After completed treatment	19.91 (2.57)	15.81–25.24	---
In present study	24.03 (4.05)	17.78–34.34	---
Curve type			
Thoracic	---	---	21 (70.0)
Thoraco-lumbar	---	---	2 (6.67)
Lumbar	---	---	7 (23.33)
Curve size of the major curve (Cobb angle)			
Before treatment	32.2 (5.59)	20–40	---
After completed treatment	37.87 (12.75)	10–70	---
In present study	45.03 (17.41)	10–97	---
Success rate at maturity*	---	---	16 (53.33)
Curve change from end of treatment to present study	7.16 (7.64)	0–27	---
Apical translation [cm]**			
Before treatment	2.05 (0.98)	0.2–4	---
After completed treatment	2.65 (1.30)	0.3–5.1	---
In present study	3.66 (1.99)	0.5–9.4	---
Rib hump angle in present study	10.37 (4.20)	4–20	---
Rib hump height in present study [cm]	3.33 (1.53)	1–7	---
Thoracic kyphosis in present study [angle]	25.33 (11.06)	9–62	---
Lumbar lordosis in present study [angle]	54.63 (10.59)	27–76	---

Note

*According to Nachemson and Peterson, the success of treatment was defined as an increase in the curve of less than 6° from the start of bracing

** the degree of the apical translation of center sacral vertical line (CSVL) according to the Harms Study Group; standard deviation (SD).

### Socio-demographic data

The mean age of patients (SG–study group) at the follow-up was 41.13 yrs. SD 3.87 (range 35–55), whereas mean age of controls (HG–healthy group) was 42.05 yrs. SD 7.41 (range 22–61). Twenty-eight females with AIS (93.4%) and 29 healthy controls (69%) were married. Of 30 female patients, 29 (96.67%) had delivered babies and the mean number of children was 2.0 SD 0.83 (range 0–4), whereas in the control group 33 females (78.57%) had delivered babies and the mean number of children in this subgroup was 1.48 SD 0.99 (range 0–3). The rate of caesarean section was 30% (9 patients) in SG and 27.3% (9 controls) in HG. Ten patients (34.48%) and 8 controls (23.53%) had experienced problems during delivery (for additional data, see Table 2).

**Table 2:** Socio-demographic characteristics of patients and healthy controls.

Characteristics	Patient group			Healthy controls			p value (ES)
	Mean (SD)	Range	N (%)	Mean (SD)	Range	N (%)	
Age at the start of treatment [yrs.]	12.43 (1.83)	10–14	---	Not applicable			
Age in present study [yrs.]	41.13 (3.87)	35–50	---	42.05 (7.41)	22–61	----	p = 0.326
Marital status							p = 0.037* (ES = 0.283)
Single	---	---	1 (3.33)	---	---	8 (19.0)	
Married	---	---	28 (93.34)	---	---	29 (69.00)	
Divorced	---	---	1 (3.33)	---	---	5 (11.9)	
Widowed	---	---	0 (0)	---	---	0 (0)	
Educational level							p = 0.004* (ES = 0.373)
Elementary	---	---	2 (6.67)	---	---	2 (4.8)	
Occupational	---	---	7 (23.33)	---	---	0 (0)	
Secondary	---	---	7 (23.33)	---	---	19 (45.2)	
University	---	---	14 (46.67)	---	---	21 (50)	
Place of residence							p = 0.001* (ES = 0.410)
Country	---	---	12 (40.0)	---	---	8 (19.0)	
Below 25 000	---	---	12 (40.0)	---	---	7 (16.7)	
Between 25 000 and 20 000	---	---	2 (6.67)	---	---	4 (9.5)	
Over 20 000	---	---	4 (13.33)	---	---	23 (54.8)	
Working time per week [hours]	33.21 (15.41)	0–60	---	42.88 (13.96)	5–70	---	p = 0.013* (ES = -0.340)
Overall working time [yrs.]	17.37 (19.13)	0–30	---	19.55 (8.94)	1–40	---	p = 0.317
No. of children	2.0 (0.83)	0–4	---	1.48 (0.99)	0–3	---	p = 0.046* (ES = 0.278)
Age at 1^st^ pregnancy**	24.86 (3.99)	19–38	---	24.50 (3.72)	18–34	---	p = 0.767
Caesarian section*	---	---	9 (30)	---	---	9 (27.3)	p = 0.745
Complications during delivery**	---	---	10 (34.48)	---	---	8 (23.53)	p = 0.344
Active hobby	---	---	13 (43.33)	---	---	16 (38.1)	p = 0.656
Active hobby per week [hours]***	3.46 (3.78)	1–15	---	5.12 (4.30)	2–20	---	p = 0.038* (ES = -0.448)

Note

* p < 0.05

**out of 29 patients and 34 participants who had delivered a child

***out of 13 patients and 17 healthy controls with an active hobby; standard deviation (SD); effect size (ES)

### Analysis of questionnaires data

Mean scores and standard deviations, the minimum, maximum, and 95% confidence intervals of SRS-22, BSSQ-Deformity, BSSQ-Brace and the SAQ were calculated and are summarized in Table 3.

**Table 3:** Descriptive statistics of the BSSQ-Brace, BSSQ-Deformity, SAQ and SRS-22 results.

Questionnaire	Patients							Healthy controls						p value (ES)
	Mean	95% CI			Min	Max	SD	Mean	95% CI		Min	Max	SD	
		from	to						from	to				
BSSQ-Deformity	7.40	9.23	12.87		4	21	4.73	Not applicable						
BSSQ-Brace	11.10	6.01	8.79		0	15	3.71	Not applicable						
SAQ														
General	3.33	3.00	3.67	1.0		5.0	0.90	2.68	2.60	3.24	2.0	5.0	1.05	p = 0.002* (ES = 0.418)
Curve	2.93	2.53	3.34	2.0		5.0	1.08	1.10	0.94	1.25	1.0	4.0	0.48	p < 0.001* (ES = 0.951)
Prominence	2.53	2.21	2.86	1.0		5.0	0.87	1.08	0.94	1.23	1.0	4.0	0.47	p < 0.001* (ES = 0.952)
Trunk shift	2.45	2.16	2.74	1.0		4.0	0.78	1.08	0.95	1.22	1.0	4.0	0.47	p < 0.001* (ES = 0.919)
Waist	3.02	2.51	3.54	1.0		5.0	1.38	1.29	1.04	1.53	1.0	5.0	0.78	p < 0.001* (ES = 0.740)
Shoulders	2.83	2.51	3.15	1.0		5.0	0.85	1.21	1.0	1.43	1.0	4.5	0.68	p < 0.001* (ES = 0.890)
Kyphosis	2.76	2.35	3.18	1.0		5.0	1.10	1.12	1.0	1.24	1.0	3.0	0.40	p < 0.001* (ES = 0.905)
Chest	2.97	2.42	3.51	1.0		5.0	1.46	1.13	1.01	1.26	1.0	3.0	0.40	p < 0.001* (ES = 0.773)
Total score	2.91	2.61	3,18	1.31		4.20	0.77	1.45	1.32	1.58	1.13	3.13	0.41	p < 0.001* (ES = 0.908)
SRS-22														
Intensity of pain	2.90	2.74	3.06	2.00		3.80	0.44	2.67	2.55	2.78	1.40	3.40	0.36	p = 0.016* (ES = 0.329)
Self-image	2.14	1.96	2.32	1.40		3.20	0.47	2.02	1.89	2.15	1.40	3.20	0.42	p = 0.249
Function/activity	2.75	2.45	3.06	1.20		4.40	0.82	1.89	1.68	2.10	1.0	3.20	0.66	p < 0.001* (ES = 0.583)
Mental health	3.05	2.89	3.20	2.00		3.80	0.41	3.00	2.89	3.11	2.40	3.8	0.35	p = 0.611
Satisfaction from treatment	2.77	2.49	3.05	1.50		4.50	0.75	Not applicable						
Total score	2.71	2.58	2.85	1.85		3.60	0.38	2.39	2.30	2.49	1.95	3.0	0.29	p < 0.001* (ES = 0.501)

Note

* p < 0.05; standard deviation (SD); confidence intervals (CI); effect size (ES); Scoliosis Research Society-22 (SRS-22); Spinal Appearance Questionnaire (SAQ); Bad Sobberheim Stress Questionnaire-Deformity (BSSQ-Deformity); Bad Sobberheim Stress Questionnaire-Brace (BSSQ-Brace).

Patients experienced a moderate level of stress connected with memories of conservative treatment; the mean value was 11.1 SD 4.73, however, the stress level related to perceived trunk deformation was high and the mean value was 7.40 SD 3.71. This difference is statistically significant (p = 0.001).

The highest number of patients (n = 14, 46.67%) experienced moderate stress related to body disfigurement. However, a similar number of patients (n = 12, 40%) reported severe stress concerning body deformity. Only 4 patients (13.33%) experienced a low stress level. Concerning the memories of stress related to completed brace treatment, 20 patients (66.7%) reported a strong stress level regarding this experience, whereas 10 females (33.33%) reported a moderate stress level.

In respect to the total scores of the SAQ, patients scored 2.91 SD 0.77. Patients exhibited the most self-criticism in the following order: General, Waist, Chest, Curve and Shoulders (average scores respectively: 3.33, 3.02, 2.97, 2.93 and 2.83). Kyphosis, Prominence and Trunk shift were the elements that are assessed the least critically by patients (average scores respectively: 2.70, 2.53 and 2.45) (Table 3).

Whereas, considering the general results of the SAQ achieved in the HG, individuals scored 1.45 SD 0.41. Healthy controls exhibited the greatest self-criticism in the following order: General, Waist, Shoulders, Chest And Kyphosis (average scores respectively: 2.68, 1.29, 1.21, 1.13, 1.12). Curve, Prominence and Trunk Shift were the least critically elements assessed by the controls (average scores respectively: 1.10, 1.08 and 1.08) (Table 3).

Table 4 shows the interpretation of answers given to open-ended questions on the SAQ. From the interpretation of answers given to question 8, it appears that most patients (n = 13 and n = 9, that is 43.33% and 26.67%) indicated rib and flank prominence, respectively, as the elements of trunk deformity most disturbing to them. (Table 3). In the HG, 39 healthy controls (92.86%) indicated that none of listed forms of body deformity bothered them the most.

**Table 4:** Distribution of results regarding SAQ questions 8, 18 and 20 in both groups studied.

Question no.	Patients	Healthy controls	p value (ES)
	n (%)	n (%)	
**Question 8: Which form of deformity bothers you the most out of these 5 categories of images?**			
None	0 (0)	39 (92.86)	p < 0.001* (ES = 0.512)
Rib prominence	13 (43.33)	0 (0)	
Flank prominence	8 (26.67)	0 (0)	
Head Chest Hips	3 (10)	3 (7.14)	
Shoulder level	3 (10)	0 (0)	
Spine prominence	3 (10)	0 (0)	
**Question 18: Of questions 9-17which are the most important to you?**			
None	12 (40.00)	34 (80.10)	p = 0.005* (ES = 0.470)
A question on the desire to have a correct trunk shape	12 (40.00)	3 (7.14)	
A question on better appearance in clothing	1 (3.33)	3 (7.14)	
A question on symmetrical hips	1 (3.33)	1 (2.38)	
A question on symmetrical breasts	2 (6.66)	0 (0)	
A question on symmetrical shoulders	1 (3.33)	1 (2.38)	
A question on surgical scar	1 (3.33)	0 (0)	
**Question 20: What would you most like to change about your body’s shape**			
Nothing	14 (46.68)	34 (80.10)	p = 0.013* (ES = 0.409)
Trunk shape	2 (6.66)	1 (2.38)	
Asymmetrical shoulders	2 (6.66)	1 (2.38)	
Rib hump	6 (20.00)	0 (0)	
Asymmetrical waist	1 (3.33)	0 (0)	
My body shape in general	5 (16.67)	6 (14.29)	

Note

* p < 0.05; effect size (ES); Spinal Appearance Questionnaire (SAQ).

From the interpretation of answers given to question 18, it appears that 12 patients (40%) would like to be more even, but another 12 patients pointed out that none of the listed items concerning body appearance was the most important to them. Interestingly, thirty-four (80.10%) healthy controls indicate that none of the listed items concerning body appearance are the most important to them.

The distribution of answers to question 20 seems interesting. It appears that as many as 14 patients (46.68%) would not change anything in their body shape, whereas 5 of them (16.67%) would like to change their body appearance in general (For details see Table 5). In the control group, most of the females (n = 34, 80.10%) would not change anything in their physical appearance.

The total score of the SRS-22 was 2.71 (SD 0.38) and 2.39 (SD 0.29) in the patient and healthy controls group, respectively (Table 2). Females from both study groups scored highest in the mental health domain (3.05 SD 0.41 in the SG and 3.0 SD 0.35 in the HG). In the HG, the worst score regards the function/activity domain (1.89 SD 0.66), whereas in the SG it regards the self-image subscale (2.14 SD 0.47).

### Comparative analyses

Tables 2 and 3 also present the results of cross-group comparisons for the socio-demographic data, general results of each scale as well as for individual domains. In terms of socio-demographic characteristics, there are statistical differences between subgroups among: educational level (p = 0.004), the Bonferroni correction revealed that study groups differ statistically in incidence of having occupational and secondary level of education at p = 0.001:, participants from SG had less often secondary than occupational level of education than participants from HG; the Bonferroni correction also revealed that study groups differ statistically in incidence of having occupational and university level of education at p = 0.003:, participants from SG had less often university than occupational level of education than healthy controls. There is also a statistical difference between subgroups among place of residence (p = 0.001): the Bonferroni correction revealed that study groups differ statistically in incidence of living in the country and a city over 20 000 inhabitants at p = 0.002, participants from SG lived more often in the country.; a Bonferroni correction also revealed that study groups differ statistically in incidence of living in a city below 25 000 inhabitants and city over 20 000 inhabitants at p = 0.001, participants from SG lived more often in in a city below 25 000 inhabitants. Results showed that subgroups statistically differ in terms of working time per week (p = 0.013, patients from SG spend less time on their occupational activity per week), no. of children (p = 0.046, patients from SG had delivered more children) and active hobby per week (p = 0.038, patients from SG spend fewer hours weekly on an active hobby). There was also revealed a statistical difference between subgroups in marital status (p = 0.037), however, a Bonferroni correction revealed that the particular comparisons between selected categories (single, married, divorced, widowed) are statistically insignificant. For details, see Table 2.

Regarding SAQ results, the study groups differ significantly in the total score and all individual domains, indicating better functioning among participants from HG. Except for the General domain (p = 0.002), the study groups differed significantly on the remaining subscales at p<0.001. Regarding the distribution of answers to open-ended questions, our study confirmed statistically significant differences for question 8, as well as questions 18 and 20 (p<0.001, p = 0.005 and p = 0.013, respectively). A Bonferroni correction revealed that, regarding question 8, the study groups differ significantly (at p < 0.0001) in incidence of pointing which form of deformity bothers them most (answer none vs rib prominence), indicating a better assessment of body shape among the healthy controls. Concerning question 18, the Bonferroni correction revealed that the study groups differ significantly (at p = 0.0005) in incidence of pointing which of questions 9–17 are the most important to participants, indicating that healthy controls more often indicated that none of questions is the most important to them, compared to indicating question no. 9 (on the desire to have a correct trunk shape) than scoliosis patients. Finally, concerning question 20, the Bonferroni correction revealed that the study groups differ significantly (at p = 0.002) in pointing what would they most like to change about their body’s shape indicating that scoliosis patients more often pointed rib hump than their body shape in general, compared to healthy controls (see Table 4).

Considering SRS-22 results, it was revealed that the SG group scored higher, signaling better functioning with reference to the pain level (p = 0.016), function/activity (p<0.001) and the total score (p<0.001) (for details see Table 3).

### Correlation between the radiographic and clinical data and patients’ well-being

Having analyzed the correlations concerning the results on specific subscales and the general results of the SAQ, after implementing a Bonferroni correction for multiple comparisons, test results whose p value exceeded the level of 0.0056, were treated as insignificant. Associations were identified between the Cobb angle after completed treatment and Prominence and Trunk Shift domains and the Total score (rs = 0.56, rs = 0.53 and rs = 0.50, respectively). In addition, significant associations were also revealed between apical translation after treatment and Shoulders and Chest domains and the Total score (rs = 0.53, rs = 0.53 and rs = 0.53, respectively) (see Table 5).

**Table 5:** Correlational analysis of patients’ characteristics and Spinal appearance questionnaire.

Characteristics	SAQ domains										
	General	Curve	Prominence	Trunk shift	Waist	Shoulders	Kyphosis		Chest		Total score
Brace application [hours/day]	rs = 0.15 p = 0.433	rs = 0.10 p = 0.613	rs = -0.17 p = 0.364	rs = 0.18 p = 1.000	rs = -0.17 p = 0.356	rs = -0.12 p = 0.537	rs = 0.15 p = 0.415		rs = -0.18 p = 0.335		rs = -0.08 p = 0.661
Brace application [months]	rs = -0.14 p = 0.460	rs = 0.20 p = 0.295	rs = -0.24 p = 0.196	rs = 0.01 p = 0.744	rs = -0.36 p = 0.051	rs = -0.19 p = 0.322	rs = -0.19 p = 0.303		rs = -0.22 p = 0.251		rs = -0.17 p = 0.367
Time after completed treatment [years]	rs = 0.10 p = 0.619	rs = -0.11 p = 0.566	rs = 0.06 p = 0.734	rs = 0.06 p = 0.328	rs = 0.32 p = 0.083	rs = 0.03 p = 0.869	rs = 0.01 p = 0.980		rs = 0.22 p = 0.253		rs = 0.19 p = 0.324
Body Mass Index											
Before treatment	rs = 0.16 p = 0.406	rs = -0.38 p = 0.041	rs = -0.23 p = 0.215	rs = -0.07 p = 0.726	rs = 0.03 p = 0.867	rs = -0.13 p = 0.506	rs = 0.02 p = 0.911		rs = -0.19 p = 0.308		rs = -0.15 p = 0.438
After completed treatment	rs = 0.07 p = 0.724	rs = -0.14 p = 0.457	rs = -0.09 p = 0.624	rs = 0.06 p = 0.734	rs = -0.18 p = 0.329	rs = -0.18 p = 0.352	rs = -0.10 p = 0.604		rs = -0.23 p = 0.216		rs = -0.13 p = 0.506
In present study	rs = 0.15 p = 0.422	rs = 0.12 p = 0.530	rs = -0.06 p = 0.768	rs = 0.13 p = 0.494	rs = -0.11 p = 0.572	rs = -0.08 p = 0.675	rs = -0.13 p = 0.489		rs = -0.06 p = 0.762		rs = 0.03 p = 0.873
Curve type	p = 0.806	p = 0.092	p = 0.153	p = 0.112	p = 0.179	p = 0.057	p = 0.026		p = 0.111		p = 0.157
Curve size of the major curve (Cobb angle)											
Before treatment	rs = 0.04 p = 0.849	rs = 0.28 p = 0.129	rs = 0.33 p = 0.175	rs = 0.33 p = 0.071	rs = 0.30 p = 0.106	rs = 0.32 p = 0.089	rs = 0.08 p = 0.661	rs = 0.42 p = 0.022		rs = 0.36 p = 0.052	
After completed treatment	rs = 0.11 p = 0.485	rs = 0.28 p = 0.127	rs = 0.56* p = 0.001	rs = 0.53* p = 0.003	rs = 0.42 p = 0.020	rs = 0.35 p = 0.056	rs = 0.07 p = 0.700	rs = 0.47 p = 0.008		rs = 0.50* p = 0.005	
In present study	rs = 0.04 p = 0.843	rs = 0.32 p = 0.084	rs = 0.46 p = 0.011	rs = 0.48 p = 0.007	rs = 0.38 p = 0.038	rs = 0.34 p = 0.066	rs = 0.06 p = 0.769	rs = 0.46 p = 0.010		rs = 0.44 p = 0.016	
Curve change from end of treatment to present study**	rs = -0.21 p = 0.259	rs = 0.17 p = 0.376	rs = -0.01 p = 0.957	rs = 0.13 p = 0.493	rs = 0.01 p = 0.959	rs = 0.05 p = 0.803	rs = 0.09 p = 0.638	rs = 0.13 p = 0.506		rs = 0.02 p = 0.906	
Apical translation [cm]***											
Before treatment	rs = 0.13 p = 0.485	rs = 0.05 p = 0.804	rs = 0.18 p = 0.354	rs = 0.01 p = 0.939	rs = 0.33 p = 0.077	rs = 0.41 p = 0.026	rs = -0.01 p = 0.961	rs = 0.43 p = 0.016		rs = 0.26 p = 0.161	
After completed treatment	rs = 0.26 p = 0.173	rs = 0.36 p = 0.053	rs = 0.40 p = 0.032	rs = 0.44 p = 0.014	rs = 0.47 p = 0.009	rs = 0.53* p = 0.002	rs = 0.11 p = 0.567	rs = 0.53* p = 0.002		rs = 0.53* p = 0.003	
In present study	rs = -0.14 p = 0.470	rs = 0.38 p = 0.036	rs = 0.24 p = 0.210	rs = 0.32 p = 0.083	rs = 0.26 p = 0.172	rs = 0.23 p = 0.220	rs = 0.18 p = 0.337	rs = 0.33 p = 0.072		rs = 0.25 p = 0.184	
Rib hump angle in present study	rs = 0.05 p = 0.785	rs = -0.01 p = 0.984	rs = 0.67 p = 0.727	rs = 0.14 p = 0.475	rs = -0.01 p = 0.944	rs = 0.01 p = 0.943	rs = 0.18 p = 0.352	rs = 0.02 p = 0.894		rs = -0.01 p = 0.965	
Rib hump height in present study [cm]	rs = 0.12 p = 0.521	rs = -0.03 p = 0.870	rs = 0.06 p = 0.740	rs = 0.16 p = 0.408	rs = 0.32 p = 0.082	rs = 0.32 p = 0.008	rs = 0.11 p = 0.592	rs = 0.34 p = 0.008		rs = 0.26 p = 0.160	
Thoracic kyphosis in present study [angle]	rs = 0.18 p = 0.326	rs = 0.16 p = 0.393	rs = 0.04 p = 0.848	rs = 0.35 p = 0.057	rs = 0.15 p = 0.432	rs = 0.23 p = 0.214	rs = 0.39 p = 0.036	rs = 0.16 p = 0.386		rs = 0.27 p = 0.156	
Lumbar lordosis in present study [angle]	rs = 0.03 p = 0.900	rs = -0.21 p = 0.272	rs = 0.05 p = 0.812	rs = 0.13 p = 0.049	rs = 0.16 p = 0.410	rs = 0.09 p = 0.632	rs = 0.05 p = 0.790	rs = 0.11 p = 0.602		rs = 0.09 p = 0.634	

Note

* p < 0.0056

**according to Nachemson and Peterson, success of treatment was defined as an increase in the curve of less than 6° from the start of bracing

*** the degree of the apical translation of center sacral vertical line (CSVL) according to the Harms Study Group; effect size (ES); Spinal Appearance Questionnaire (SAQ).

Considering the associations between BSSQ, SRS-22 and radiographic and clinical data, after implementing a Bonferroni correction for multiple comparisons, test results whose p value exceeded the level of 0.0063, were treated as insignificant. Finally, we did not reveal any significant associations. For details, see Table 6.

**Table 6:** Correlational analysis of clinical and radiological patients’ characteristics, SRS-22 and BSSQ.

Characteristics	SRS-22							BSSQ- Brace	BSSQ-Deformity
	Intensity of pain		Self-image	Function/ activity	Mental health	Satisfaction with treatment	Total score		
Brace application [hours/day]	rs = 0.12 p = 0.515		rs = 0.33 p = 0.078	rs = 0.30 p = 0.104	rs = 0.18 p = 0.334	rs = 0.11 p = 0.580	rs = 0.31 p = 0.097	rs = 0.21 p = 0.274	rs = -0.21 p = 0.275
Brace application [months]	rs = 0.23 p = 0.226		rs = -0.02 p = 0.920	rs = 0.20 p = 0.282	rs = -0.21 p = 0.269	rs = -0.28 p = 0.129	rs = -0.01 p = 0.969	rs = -0.05 p = 0.812	rs = -0.02 p = 0.906
Time after completed treatment [years]	rs = -0.29 p = 0.122		rs = 0.06 p = 0.763	rs = -0.02 p = 0.908	rs = -0.02 p = 0.912	rs = 0.09 p = 0.655	rs = 0.01 p = 0.992	rs = 0.20 p = 0.279	rs = 0.09 p = 0.653
Body Mass Index									
Before treatment	rs = -0.02 p = 0.944		rs = -0.20 p = 0.282	rs = -0.39 p = 0.032	rs = -0.35 p = 0.062	rs = -0.29 p = 0.126	rs = -0.45 p = 0.012	rs = 0.15 p = 0.438	rs = 0.16 p = 0.391
After completed treatment	rs = 0.29 p = 0.119		rs = -0.16 p = 0.384	rs = -0.19 p = 0.307	rs = -0.23 p = 0.214	rs = -0.40 p = 0.031	rs = -0.28 p = 0.138	rs = 0.25 p = 0.178	rs = 0.26 p = 0.158
In present study	rs = 0.16 p = 0.384		rs = -0.11 p = 0.552	rs = -0.06 p = 0.769	rs = -0.12 p = 0.545	rs = -0.19 p = 0.321	rs = -0.12 p = 0.531	rs = 0.24 p = 0.209	rs = 0.10 p = 0.587
Curve type	p = 0.750		p = 0.090	p = 0.353	p = 0.492	p = 0.436	p = 0.218	p = 0.787	p = 0.174
Curve size of the major curve (Cobb angle)									
Before treatment	rs = -0.05 p = 0.776		rs = -0.16 p = 0.392	rs = 0.15 p = 0.419	rs = 0.01 p = 0.980	rs = 0.07 p = 0.711	rs = -0.11 p = 0.560	rs = -0.21 p = 0.277	rs = -0.22 p = 0.248
After completed treatment	rs = 0.01 p = 0.961		rs = 0.16 p = 0.395	rs = 0.29 p = 0.119	rs = -0.04 p = 0.818	rs = 0.25 p = 0.168	rs = 0.29 p = 0.126	rs = -0.11 p = 0.553	rs = -0.24 p = 0.211
In present study	rs = 0.06 p = 0.760		rs = -0.13 p = 0.133	rs = 0.24 p = 0.197	rs = 0.03 p = 0.864	rs = 0.22 p = 0.239	rs = 0.26 p = 0.161	rs = -0.10 p = 0.583	rs = -0.28 p = 0.127
Curve change from end of treatment to present study*	rs = -0.01 p = 0.961		rs = 0.25 p = 0.183	rs = 0.02 p = 0.927	rs = 0.24 p = 0.206	rs = 0.09 p = 0.624	rs = 0.18 p = 0.535	rs = -0.14 p = 0.990	rs = -0.01 p = 0.455
Apical translation [cm]**									
Before treatment	rs = 0.07 p = 0.701	rs = -0.07 p = 0.718		rs = -0.02 p = 0.922	rs = 0.01 p = 0.953	rs = 0.12 p = 0.521	rs = 0.02 p = 0.905	rs = -0.26 p = 0.166	rs = -0.22 p = 0.243
After completed treatment	rs = -0.01 p = 0.938	rs = 0.22 p = 0.395		rs = 0.19 p = 0.315	rs = 0.22 p = 0.234	rs = 0.31 p = 0.093	rs = 0.26 p = 0.173	rs = -0.13 p = 0.509	rs = -0.32 p = 0.087
In present study	rs = -0.37 p = 0.044	rs = 0.29 p = 0.114		rs = 0.03 p = 0.864	rs = 0.20 p = 0.291	rs = -0.44 p = 0.016	rs = 0.15 p = 0.416	rs = -0.11 p = 0.552	rs = -0.29 p = 0.120
Rib hump angle in present study	rs = -0.22 p = 0.233	rs = 0.06 p = 0.820		rs = -0.26 p = 0.790	rs = 0.15 p = 0.424	rs = 0.19 p = 0.315	rs = -0.11 p = 0.552	rs = 0.08 p = 0.687	rs = -0.36 p = 0.053
Rib hump height in present study [cm]	rs = -0.29 p = 0.117	rs = 0.04 p = 0.744		rs = -0.32 p = 0.090	rs = 0.15 p = 0.441	rs = 0.15 p = 0.420	rs = -0.19 p = 0.317	rs = -0.01 p = 0.991	rs = -0.20 p = 0.280
Thoracic kyphosis in present study [angle]	rs = -0.024 p = 0.899	rs = -0.11 p = 0.548		rs = -0.05 p = 0.173	rs = 0.04 p = 0.842	rs = 0.11 p = 0.551	rs = -0.02 p = 0.902	rs = -0.13 p = 0.510	rs = -0.15 p = 0.440
Lumbar lordosis in present study [angle]	rs = -0.07 p = 0.730	rs = 0.08 p = 0.680		rs = -0.22 p = 0.246	rs = 0.24 p = 0.195	rs = 0.03 p = 0.869	rs = -0.01 p = 0.994	rs = 0.01 p = 0.953	rs = -0.22 p = 0.235

Note

*according to Nachemson and Peterson, success of treatment was defined as an increase in the curve of less than 6° from the start of bracing

**the degree of the apical translation of center sacral vertical line (CSVL) according to the Harms Study Group; Scoliosis Research Society-22 (SRS-22); Bad Sobberheim Stress Questionnaire-Deformity (BSSQ-Deformity); Bad Sobberheim Stress Questionnaire-Brace (BSSQ-Brace).

### Correlation between the socio-demographic data and patients’ well-being

After implementing a Bonferroni correction for multiple comparisons, test results whose p value exceeded the level of 0.0056, were treated as insignificant. Finally, we did not reveal any significant associations. For details, see Table 7.

**Table 7:** Associations between the socio-demographic data and questionnaire results.

	Age at the present [yrs.]	Age at the start of treatment[yrs.]	Marital status	Educational level	Place of residence	Working time per week [hours]	Overall working time [yrs.]	No. of children	Active hobby per week [hours]
BSSQ-Deformity	rs = 0.11 p = 0.569	rs = 0.27 p = 0.143	p = 0.331	p = 0.273	p = 0.945	rs = 0.12 p = 0.827	rs = 0.10 p = 0.595	rs = -0.06 p = 0.738	rs = -0.08 p = 0.790
BSSQ-Brace	rs = 0.16 p = 0.391	rs = -0.03 p = 0.890	p = 0.716	p = 0.382	p = 0.151	rs = 0.05 p = 0.584	rs = 0.05 p = 0.792	rs = 0.13 p = 0.510	rs = 0.44 p = 0.129
SAQ									
General	rs = 0.06 p = 0.758	rs = 0.14 p = 0.476	p = 0.216	p = 0.936	p = 0.643	rs = -0.12 p = 0.042	rs = -0.16 p = 0.403	rs = -0.17 p = 0.358	rs = 0.04 p = 0.898
Curve	rs = -0.10 p = 0.613	rs = -0.44 p = 0.015	p = 0.496	p = 0.778	p = 0.781	rs = 0.34 p = 0.026	rs = 0.13 p = 0.491	rs = -0.13 p = 0.486	rs = -0.25 p = 0.415
Prominence	rs = 0.05 p = 0.813	rs = -0.04 p = 0.845	p = 0.326	p = 0.480	p = 0.145	rs = -0.02 p = 0.878	rs = 0.06 p = 0.714	rs = 0.38 p = 0.039	rs = 0.14 p = 0.641
Trunk shift	rs = 0.22 p = 0.246	rs = -0.08 p = 0.693	p = 0.219	p = 0.186	p = 0.520	rs = 0.13 p = 0.425	rs = 0.13 p = 0.506	rs = 0.04 p = 0.818	rs = 0.17 p = 0.641
Waist	rs = 0.21 p = 0.255	rs = 0.24 p = 0.194	p = 0.119	p = 0.172	p = 0.080	rs = 0.04 p = 0.805	rs = 0.08 p = 0.668	rs = 0.01 p = 0.095	rs = 0.31 p = 0.306
Shoulders	rs = -0.05 p = 0.791	rs = -0.03 p = 0.887	p = 0.313	p = 0.742	p = 0.468	rs = -0.06 p = 0.773	rs = 0.08 p = 0.656	rs = -0.01 p = 0.968	rs = 0.05 p = 0.885
Kyphosis	rs = 0.08 p = 0.684	rs = -0.07 p = 0.694	p = 0.227	p = 0.621	p = 0.598	rs = -0.02 p = 0.906	rs = 0.18 p = 0.340	rs = -0.06 p = 0.742	rs = -0.20 p = 0.510
Chest	rs = 0.11 p = 0.580	rs = 0.02 p = 0.908	p = 0.312	p = 0.700	p = 0.402	rs = -0.09 p = 0.588	rs = 0.18 p = 0.347	rs = -0.07 p = 0.716	rs = 0.04 p = 0.897
Total score	rs = 0.11 p = 0.554	rs = -0.01 p = 0.974	p = 0.125	p = 0.356	p = 0.287	rs = -0.03 p = 0.827	rs = 0.08 p = 0.682	rs = -0.01 p = 0.939	rs = 0.15 p = 0.620
SRS-22									
Intensity of pain	rs = -0.32 p = 0.081	rs = -0.16 p = 0.411	p = 0.341	p = 0.499	p = 0.090	rs = 0.05 p = 0.821	rs = -0.25 p = 0.185	rs = 0.36 p = 0.052	rs = -0.01 p = 0.974
Self-image	rs = -0.02 p = 0.896	rs = -0.09 p = 0.636	p = 0.367	p = 0.870	p = 0.353	rs = 0.12 p = 0.570	rs = 0.08 p = 0.680	rs = -0.08 p = 0.662	rs = 0.42 p = 0.149
Function/activity	rs = -0.19 p = 0.304	rs = -0.43 p = 0.017	p = 0.164	p = 0.968	p = 0.659	rs = 0.12 p = 0.558	rs = -0.02 p = 0.908	rs = 0.07 p = 0.728	rs = -0.34 p = 0.260
Mental health	rs = -0.05 p = 0.785	rs = -0.21 p = 0.273	p = 0.986	p = 0.644	p = 0.523	rs = -0.14 p = 0.518	rs = 0.10 p = 0.608	rs = 0.22 p = 0.249	rs = 0.30 p = 0.313
Satisfaction from treatment	rs = 0.10 p = 0.601	rs = 0.01 p = 0.965	p = 0.117	p = 0.609	p = 0.518	rs = -0.06 p = 0.785	rs = 0.22 p = 0.242	rs = -0.19 p = 0.303	rs = 0.35 p = 0.246
Total score	rs = -0.13 p = 0.485	rs = -0.33 p = 0.074	p = 0.285	p = 0.979	p = 0.590	rs = 0.07 p = 0.745	rs = -0.01 p = 0.982	rs = 0.15 p = 0.414	rs = 0.18 p = 0.557

*Note* Scoliosis Research Society-22 (SRS-22); Spinal Appearance Questionnaire (SAQ); Bad Sobberheim Stress Questionnaire-Deformity (BSSQ-Deformity); Bad Sobberheim Stress Questionnaire-Brace (BSSQ-Brace).

### Associations between questionnaire results

As seen in Table 8, after implementing a Bonferroni correction for multiple comparisons, test results whose p value exceeded the level of 0.0125, were treated as insignificant. It was revealed that only BSSQ-Deformity displays a significant correlation with SRS-22 (rs = -0.48) (see Table 8).

**Table 8:** Associations between SRS-22. SAQ, BSSQ-Deformity and BSSQ-Brace.

Study questionnaires	BSSQ-Deformity	BSSQ-Brace	SAQ Total score	SRS-22 Total score
BSSQ-Deformity	---	rs = 0.32 p = 0.088	rs = -0.42 p = 0.020	rs = -0.48* p = 0.010
BSSQ-Brace	rs = 0.32 p = 0.088	---	rs = -0.27 p = 0.156	rs = -0.08 p = 0.667
SAQ Total score	rs = -0.42 p = 0.020	rs = -0.27 p = 0.156	---	rs = 0.47 p = 0.018
SRS-22 total score	rs = -0.48* p = 0.010	rs = -0.08 p = 0.667	rs = 0.47 p = 0.018	---

Note

* p < 0.0125; Scoliosis Research Society-22 (SRS-22); Spinal Appearance Questionnaire (SAQ); Bad Sobberheim Stress Questionnaire-Deformity (BSSQ-Deformity); Bad Sobberheim Stress Questionnaire-Brace (BSSQ-Brace).

## Discussion

A number of long-term follow-up studies on AIS have been published [2–5,33–34]. Many of those studies only focus on one topic, mostly on radiological findings in one plane, with no comparison group of straight individuals. A non-consecutive patient series and a high number of dropouts in these studies limit the conclusions that can be drawn [34].

Bearing in mind the results of these studies, we assumed that monitoring the functioning of adult females with AIS should be routinely implemented after brace treatment is completed. The longitudinal exploration of the perception of disease and possible psychopathological implications would allow determining useful practical implications for the clinicians. In addition, we believe that long-term follow-up studies can provide reliable information for patients who will undergo conservative or surgical treatment due to AIS.

To our knowledge, this is the first study of long-term effects after more than two decades of completed Milwaukee brace treatment, concerning patient’s complex evaluation of disfigurement by means of trunk profiles depicting various degrees of scoliosis-related trunk deformity, or memories of stress experienced during wearing the brace and regarding body deformity. A control group of randomly selected from a larger sample healthy females, assembled for comparison purposes, enabled a comprehensive assessment of scoliosis patients, and, owing to this fact, our study results add to the complexity of long post-treatment evaluations of brace treatment in AIS. It was confirmed that adult scoliosis patients, despite over 20 years after completed Milwaukee brace treatment, are further concerned about their appearance, when questioned about nonverbal assessment of e.g. their own shoulder level, waist asymmetry, body curve or rib prominence. At the same time, emotional tension regarding the experience of brace-wearing was reported at a very high level. Moreover, adult patients questioned about their memories of emotional tension, reported higher stress levels due to brace application when compared to the stress related to trunk deformation alone.

Considering the issue of progression of scoliosis, Negrini et al. [35] summarized the results of conservative treatment. They referred to eg Weinstein et al. [36] who found that the rate of success (curves remaining below 50 degrees) was 38/51 in the brace group and 27/65 in the observation group. The results were in favor of brace. In addition, they indicated Coillard et al. [37] work, in which the authors reported the rate of success (correction or stabilization, recognized as 58 degrees or less of curve progression) as 21/26 in the brace group and 9/21 in the control group. The results were also in favor of brace. Those data are consistent with results of Milwaukee brace treatment referred in the current study and enters at issue of natural differential effects of conservative treatment due to adolescent idiopathic scoliosis.

Questions arise as to the long-term effects of bracing on the psyche, on the curve and on the everyday activity of adult scoliosis patients. In our opinion, such variables as social functioning, satisfaction with appearance, feeling of attractiveness, or stress level regarding body deformation also required more in-depth investigation in adult scoliosis populations. In a similar study, Noonan et al. [38] evaluated the psychosocial characteristics of patients treated for AIS at an average follow-up of 7 years. They found that patients' perceptions of discrimination and a lower satisfaction with their overall appearance was recalled during the brace treatment phase, but on reaching adulthood there were no more differences in those characteristics in patients compared with healthy controls. Interestingly, in their study concerning scoliosis patients at least 20 years after treatment, Danielsson et al. [7] indicated the mean curve in the long-term follow-up was slightly increased when compared with the original curves and the curve after weaning. In addition, no major impact on patients’ HRQoL was observed. Furthermore, Gabos et al. analyzed long-term outcomes in females with AIS who had been treated with the Wilmington orthosis and indicated that 93% of them reported no subjective deterioration in their physical appearance, the cosmetic appearance of the back, or their self-image in the period since they discontinued using the brace [39].

Our study findings partly confirm the outcomes referred to by e.g. Danielsson et. Al, Noonan et. al, or Gabos et al. [7,38,39], since in view of SRS-22 results, adult patients scored surprisingly higher in the intensity of pain and function/activity domains, as well as in the total score, indicating even better functioning in those areas. Those findings might result from recommendations for scoliosis patients during and after completed brace treatment by doctors. Those recommendations focus mainly on physical activity and/or participating in rehabilitation programmes. However, we did not observe any discrepancies concerning the remaining domains, such as mental health or self-image domains. This indicates that our study hypothesis, regarding SRS-22 findings, could not be supported.

Fällström et al. [40], in a series of 157 patients treated surgically and/or with Milwaukee braces, revealed that 9 years after treatment was completed, one-half of the brace group had definite signs of a negative body image concept. Those data are consistent with results derived from our study by means of the SAQ that the experience of wearing the Milwaukee brace has a long-term negative effect on patients’ perceptions of their appearance, including particular aspects of scoliosis-related deformity, e.g. curve, prominence, kyphosis, chest, shoulder level or waist asymmetry. Those results supported our primary assumption.

However, some interesting and apparently contradictory results concerning self- and body-image assessment in scoliosis patients must be discussed. As pointed out above, the study groups do not differ regarding the SRS-22 self-image domain, but, at the same time, significant discrepancies relating to the total score and all individual domains of the SAQ have been confirmed. A possible explanation of such an inconsistency might be that SRS-22 represents the traditional verbal assessment of the patient’s feeling of attractiveness and appearance, such as their appearance in clothes, or influence of body appearance on personal relationships, whereas the SAQ provides trunk profiles depicting various degrees of body deformity, which gives patients a direct, nonverbal assessment of e.g. their own shoulder level, waist asymmetry, body curve or rib prominence. In addition, the lowest score among SRS-22 domains regards the self-image subscale. Furthermore, as our study presents the first long-term evaluation of body image in Milwaukee brace-treated adult patients by means of the SAQ, we cannot compare results derived from current study to similar patients’ evaluations, also performed by means of SAQ. In addition, some explanation of those study results might question the validity of SAQ and SRS-22 in particular when used in healthy individuals. On the other hand, many authors, e.g. Berven et al., Lonner et al. or Chaib et al. [41–43] determined the effect of spinal deformity on patients quality of life by means of comparison of results of SRS-22 in adolescent idiopathic scoliosis, and healthy controls. For example, Berven et al. [41] determined the validity and reliability of the modified SRS-22 for use in the assessment of deformity in adults and demonstrated good discriminate validity of the SRS-22 in differentiating between affected and unaffected adults, whereas Chaib et al. [43] analyzed postoperative perceived health status in adolescent following idiopathic scoliosis surgical treatment by means of French SRS-22, which was also completed by healthy controls.

Especially interesting results were obtained when adult scoliosis patients were questioned about their memories of emotional tension experienced during brace treatment and concerning scoliosis-related deformity. It must be emphasized that body deformities related to scoliosis, such as rib hump or decomposition of the trunk, are sources of stress and fear and disturb the development of body image. At the same time, many authors, e.g. Clayson et al. [44], emphasize that the necessity of wearing an orthopedic brace may cause the patient emotional distress. They highlight the fear that patients have regarding using a brace in relation to their social life. Our study results indicated most of the patients had experienced moderate or severe stress related to body disfigurement and regarding the Milwaukee brace treatment implemented in puberty, which confirms our primary hypothesis. Furthermore, our results are in accordance to observations of Botens-Helmut et al. and Kotwicki et al. [29,45] regarding higher stress levels due to brace application when compared to the stress related to trunk deformation alone. This suggests that patients experiencing stress related to body disfigurement often experience additional stress related to conservative treatment.

Considering correlations between the evaluation of body appearance and scoliosis parameters, such as Cobb angle, Asher et al. reported that the trunk deformity during the last control visit did not correlate with the treatment satisfaction level [46]. Haher et al. also reported that the radiological status did not correlate with the satisfaction level [47]. Benli et al. pointed out that patients had high treatment-satisfaction scores, irrespective of their final curve patterns, and all with a neurological deficit, except for one patient, said ‘‘yes” when asked if they would have accepted the same course of treatment [48].

Interestingly, contrary to those reports, our study revealed significant associations between the nonverbal evaluation of body appearance and Cobb angle after completed treatment, or apical translation after completed treatment. Our study findings are of special importance, since, as indicated in many previous studies, negligible or weak relationships exist between the size or severity of disfigurement and psychological dysfunction [49]. In addition, we did not reveal any significant associations between the duration of the follow-up period or brace-wearing-related data.

Dyl et al. and Ferguson et al. [50,51] indicated that clinically significant body image concerns are associated with higher levels of depression, anxiety or mood disorders in general. In addition, dissatisfaction with body image may play a significant role in the development of low self-esteem, emotional problems and depression [52]. Therefore, we assumed the emotional tension regarding disfigurement or conservative treatment, may be related to patients’ assessment of spinal appearance. However, our study results did not support the evidence of relationships between patients’ perception of body image, as measured by the SAQ, and stress level due to body deformity.

One of the greatest concerns for females with AIS is whether they will be able to give birth through a normal delivery [53,54]. The finding that scoliosis patients do not differ significantly in terms of sociodemographic measures was also reported in previously published studies [55,56]. Danielsson and Nachemson reported long-term outcomes regarding childbearing and sexual life in AIS women, compared with matched control subjects who did not have scoliosis [8] and found that patients appeared to function well with regard to marital status and number of children. In addition, Danielsson et al [7] revealed no major impact of scoliosis on marriage, childbearing, or the degree of physical strain during work or leisure time. Similarly, like Benli et al. [48], in a group of patients treated conservatively, we summarized that scoliosis and brace treatment regimen did not affect patients’ marital status or childbearing negatively. However, significant differences with regard to educational levels, hours spent on the occupational activity and active hobby per week in favor of the healthy controls were confirmed.

### Clinical relevance

The results of our study indicated that the patients’ quality of life, as measured by the SRS-22, is associated with levels of emotional distress due to body disfigurement. It is particularly important as far as practical implications are concerned, since the emotional distress might constitute a potential risk leading to decrease of patients’ general well-being. It ought to be one of the factors taken into account when considering psychological screening and in providing appropriate support for AIS females.

It was also revealed that entire domain of patients treated nonoperatively with the Milwaukee brace, when followed into middle age, is more concerned about body appearance, but less with back pain and everyday activities, than healthy females. Nevertheless, patients must be aware that bigger curves after completed treatment might still be associated in the future with more concerns about their appearance and satisfaction with treatment results. Thus, those patients should be routinely supported by means of group or individual session, since their positive influence in the prevention of psychosocial impairment has been confirmed [57].

To summarize, it is necessary to carefully investigate the emotional burden of adult females with AIS experience due to spinal disfigurement and orthosis wearing, to provide them with the appropriate individual psychological support and reliable information for patients qualified to undergo conservative treatment.

### Study limitations

There are some limitations to the present study. Firstly, in view of the fact that validated scoliosis-specific assessment tools were not available 20 years ago, long-term assessment of conservative treatment of AIS must be retrospective. Secondly, the response rate was relatively low. Our study group consisted of 30 study participants. However, this is expected in a study with a very long-term follow-up assessment. Thirdly, as the patient group and the controls were different in number of children, working time, and living area, the differences in the results of the questionnaires of this study might not be due to the presence of scoliosis alone. In addition, we are aware that those differences might have influenced the study results in regards to e.g. body image disturbances or mental health. To sum up, further studies with a longer follow-up and in a group of patients treated surgically for comparative purposes in terms of nonverbal assessment of spinal appearance and trunk deformity-related emotional stress are needed to expand the results of our research.

## Conclusion

The findings of the current study add to the complexity of long follow-up evaluations of AIS, amongst females treated with a Milwaukee brace. We conclude that long-term results were not conclusive regarding the nonverbal assessment of body image and emotional tension regarding the experiences of brace-wearing. In addition, future patients can be reassured that scoliosis does not negatively affect everyday activities, pain level, childbearing and mental health. However, the subjects in our study who declared to have psychological problems due to scoliosis had bigger curve size after completed treatment.

## Supporting information

S1 File Study participants data and SRS-22, SAQ, BSSQ-Brace and BSSQ-Deformity. (XLS)
